# Study protocol for the translating research in elder care (TREC): building context through case studies in long-term care project (project two)

**DOI:** 10.1186/1748-5908-4-53

**Published:** 2009-08-11

**Authors:** Jo Rycroft-Malone, Sue Dopson, Lesley Degner, Alison M Hutchinson, Debra Morgan, Norma Stewart, Carole A Estabrooks

**Affiliations:** 1 Centre for Health-Related Research, Bangor University, Bangor, UK; 2 Said Business School, University of Oxford, Oxford, UK; 3 Faculty of Nursing, University of Manitoba, Winnipeg, MB, Canada; 4 Faculty of Nursing, University of Alberta, Edmonton AB, Canada; 5 Canadian Centre for Health & Safety in Agriculture, University of Saskatchewan, Saskatoon, SK, Canada; 6 College of Nursing, University of Saskatchewan, Saskatoon, SK, Canada

## Abstract

**Background:**

The organizational context in which healthcare is delivered is thought to play an important role in mediating the use of knowledge in practice. Additionally, a number of potentially modifiable contextual factors have been shown to make an organizational context more amenable to change. However, understanding of how these factors operate to influence organizational context and knowledge use remains limited. In particular, research to understand knowledge translation in the long-term care setting is scarce. Further research is therefore required to provide robust explanations of the characteristics of organizational context in relation to knowledge use.

**Aim:**

To develop a robust explanation of the way organizational context mediates the use of knowledge in practice in long-term care facilities.

**Design:**

This is longitudinal, in-depth qualitative case study research using exploratory and interpretive methods to explore the role of organizational context in influencing knowledge translation. The study will be conducted in two phases. In phase one, comprehensive case studies will be conducted in three facilities. Following data analysis and proposition development, phase two will continue with focused case studies to elaborate emerging themes and theory. Study sites will be purposively selected. In both phases, data will be collected using a variety of approaches, including non-participant observation, key informant interviews, family perspectives, focus groups, and documentary evidence (including, but not limited to, policies, notices, and photographs of physical resources). Data analysis will comprise an iterative process of identifying convergent evidence within each case study and then examining and comparing the evidence across multiple case studies to draw conclusions from the study as a whole. Additionally, findings that emerge through this project will be compared and considered alongside those that are emerging from project one. In this way, pattern matching based on explanation building will be used to frame the analysis and develop an explanation of organizational context and knowledge use over time.

An improved understanding of the contextual factors that mediate knowledge use will inform future development and testing of interventions to enhance knowledge use, with the ultimate aim of improving the outcomes for residents in long-term care settings.

## Background

In this issue of Implementation Science, we present a series of three study protocols: an overview of the Translating Research in Elder Care (TREC) program [1]; TREC project one (Study Protocol for Translating Research in Elder Care: Building Context – an Organizational Monitoring Program in Long-Term Care Project ) [2]; and TREC project two (Study Protocol for Translating Research in Elder Care: Building Context through Case Studies in Long-Term Care Project). The purpose of this paper is to report the study protocol for Project 2.

Current thinking and research findings suggest that the context of healthcare organizations can mediate the use of knowledge in practice. However, little is known about how this occurs [3-10]. There is evidence to show that a number of factors might make an organizational context more conducive to change [4,11]. These include components that are both identifiable and potentially modifiable. Further research is required to determine how and why these factors influence organizational context and knowledge use. An improved understanding of organizational context and its relationship to knowledge use in the nursing home sector should provide direction for designing and testing interventions to improve outcomes. Improved outcomes are desirable from the perspectives of residents and their families who have to deal with the difficult sequelae of the effects of aging; the facilities and their staff who are committed to providing high quality care; and the society at large that values the lifetime contributions that elders have made.

Broadly, findings from research show that knowledge translation is a complex, non-linear process involving multiple factors and interactions. Supported by findings from a case study meta-analysis [4], multi-site, multi-disciplinary research from evidence-into-practice projects show that a number of factors may be influential in the translation of knowledge into practice [3,6-8,10]. Specifically in relation to this study, organizational context is emerging as a potentially potent mediator of the implementation of evidence into practice. However, despite a growing evidence base, we still do not know if some contextual factors are more influential than others, or how they operate and interrelate to result in conditions more conducive to knowledge translation.

It has been argued that the organizational context in which knowledge translation takes place should be conceptualized as multi-dimensional, dynamic, and multi-layered [4,12,13]. As such, a number of potentially influential contextual factors at micro-, meso-, and macro-levels are emerging from evidence-based practice, research utilization, diffusion of innovations, and quality improvement bodies of literature. These include 'hard' factors, such as availability and accessibility of resources [14,15] and 'soft' factors, such as culture [12,16-18], power [4,12], leadership [19-21], organizational support [22,23], team climate [24], and structural factors [25]. Specifically, Sheldon *et al*.'s [26] evaluation of United Kingdom uptake of national guidelines found that healthcare organizations that were financially stable and had strong governance functions were more likely to adopt guidance than those without these features. Additionally, individuals and teams can play positive and negative roles in knowledge translation, which includes the influence of professional and social networks [4,27]. Researchers exploring nurses' use of evidence-based clinical guidelines in practice have identified leadership as a facilitator of their sustained use [19,20]. These findings showed that leaders supported colleagues to change practice in line with guideline recommendations, created a vision for evidence-based practice, and influenced regulatory factors to make guideline use easier. Significantly, leaders were present at all levels of the organization, including at the frontline and executive level in various positions, such as champions, advanced practice nurses, managers, and executives. Furthermore, capacities, such as organizational learning, knowledge management, and communities of practice [28-30] have also been identified as possibly key to developing the potential for sustained use of knowledge in practice.

There is still much to learn about the influence of organizational context and contextual factors in the use of evidence in practice. Specifically, to date, the majority of knowledge translation research has taken place in acute care settings. Therefore, little is known about what contextual factors may influence knowledge translation in long-term care settings, or how these may affect knowledge use. Additionally, previous research in which contextual factors have been identified as influential has been conducted in settings with mainly registered/regulated staff. As such, we do not know whether and how organizational context affects the practice and use of knowledge by non-registered/regulated staff. Furthermore, previous research has been conducted as one-off and/or retrospective evaluations, which provide a 'snapshot' of organizational context rather than a longitudinal view.

### Purpose of this study

The overall purpose of this study is to develop a robust explanation of the way that organizational context mediates the use of knowledge in practice in long-term care facilities.

### Objectives

Specific objectives:

1. To describe the key factors that constitute organizational context as it affects the use of knowledge in practice.

2. To describe the relationship and interactions among these factors.

3. To describe the social and professional networks that affect knowledge use.

4. To describe the role of key actors in knowledge use at various levels of the organization.

5. To describe how history has shaped the development of the organizational context as it relates to knowledge use.

6. To describe how organizational contexts develop and change over time.

7. To demonstrate the relationship between key factors of organizational context relative to knowledge use and resident outcomes (through linkage to project one data) [2].

## Methods

### Design

This case study project has been designed as longitudinal qualitative work using exploratory and interpretive methods. The project will be undertaken in two phases across the Canadian Prairie Provinces; Alberta, Saskatchewan, and Manitoba. Phase one will involve three comprehensive case studies that will be followed by phase two involving additional focused case studies to facilitate elaboration of emerging themes about the relationship between the long-term care facilities' contexts and knowledge use in practice.

Key decisions shaping case study research are: the decision about how many cases are to be studied and the role of comparison; the timeframe adopted (that is, a cross-sectional or snapshot approach versus a longitudinal investigation; the theory to guide the analysis; and the extent to which organizational context is subject to analysis. Our team has thought carefully and critically about these issues, and has designed our approach as follows.

### Approach

Case study research involves drawing on multiple sources of evidence to understand a semi-bounded phenomena (*i.e*., knowledge translation) within its real life context (*i.e*., long-term care). This approach relies on multiple sources of evidence [31] and frequently employs both quantitative and qualitative methods. As case study legitimizes an eclectic, pragmatic approach to the conduct of research we will be drawing on three complementary methodologies; ethnography (data collection methods), grounded theory (analysis and theory development), and participatory action research (working with site participants to develop site specific approaches to data collection).

The research objectives require descriptive and explanatory case study work in order to describe how organizational context influences knowledge translation in long-term care settings. However, data about cause and effect relationships are also required in order to explain which causes produce which effects in relation to knowledge translation [32]. In order to fully illuminate the research questions and assist in explanation building and transferability of findings, multiple cases will be included.

The Promoting Action on Research Implementation in Health Services (PARIHS) framework is the theoretical framework underpinning TREC [33-35]. The framework has been theoretically and empirically developed to represent the interplay and interdependence of the many factors influencing the successful translation of knowledge into practice; explained by a function of the relation between evidence, context and facilitation [5,12,34].

The framework, which underpins TREC as a whole, is particularly relevant to this study because:

1. It will provide a conceptual guide for mapping the contextual factors influencing knowledge translation in long-term care settings at various levels.

2. Understanding the role of organizational context in knowledge translation is the main purpose of this study. Both the conceptual framework and methodological approach will acknowledge and value the role of organizational context and its component parts (*e.g*., culture, leadership, evaluation) in knowledge translation.

3. It facilitates the gathering of individual (*e.g*., practitioner and resident) experiences, as well as appreciating the fit with the broader context of care delivery.

Method triangulation will be used to enhance the credibility and transferability of the conclusions drawn from the data. The unique characteristics of the different data collection methods will allow a more comprehensive understanding of organizational context and knowledge translation to emerge. Data collection methods will be used within each study site. While the data collection methods will be the same in the comprehensive and focused case study sites, comprehensive case study site data collection will be more in-depth than the focused sites by virtue of the fact that a greater amount of time will be spent in the sites.

### Cases – definition

A 'case' is being defined as a particular long-term care setting and the 'embedded units' [32] – knowledge translation practices in relation to falls, pain management, behavior management, and skin care. In this way, knowledge translation activities and behaviors will be studied in the real life of the practice context and their impact more easily evaluated.

**Comprehensive case studies **(n = 3) will be conducted in each of Alberta, Saskatchewan, and Manitoba over a six-month period. This will involve up to one month spent conducting information sessions to familiarize staff with the purpose and procedures of the study. This will be followed by approximately one month in each facility for intensive observation and interview data collection. In month three, the researchers will undertake preliminary data analysis, and then in month four they will return to the field to confirm emerging findings through interviews. The process of data analysis will be repeated in month five, and the researchers will again return to the field in month six to verify their findings through group discussions with staff in naturally-occurring meetings. During months four to six the researchers will also undertake document analysis. The total amount of time the researchers spend in the field will be a maximum of six months. We will re-enter the sites a year later to observe any changes in organizational context. This will involve up to one month of non-participant observation and interviews with selected individuals identified by the researchers as key informants (phase one is currently underway).

Focused case studies will be conducted in selected settings to allow elaboration of emerging themes about the relationship between the organizational context within long-term care facilities and knowledge use in practice. Data collection in these sites will occur over two to four months and will comprise one month spent conducting information sessions to familiarize staff with the purpose and procedures of the study. This will be followed by up to one month of non-participant observation and interviews, which will then be followed by interviews with staff and possibly family members.

### Sampling

#### Phase one

One comprehensive case study in each province will be conducted (Alberta, Saskatchewan, and Manitoba). Sites will be purposively sampled. The comprehensive cases in phase one will be selected from the list of 30 urban facilities being sampled for project one [2]. For pragmatic reasons (*e.g*., travel distance) only urban sites are to be included in phase one. The modal type of facility in terms of key characteristics (*e.g*., size, operational model) for each province will be purposively selected from the list of facilities selected for project one. Modal facilities will be approached, and in consideration of the following criteria, will be selected to participate:

1. Interest and willingness among management to grant access for the study.

2. Minimal level of organizational flux.

3. Willingness of frontline managers, healthcare aides, and other staff to be observed and interviewed for the study.

4. Practical issues, such as travel time to the facility.

5. Opportunities to maximize data collection by the existence of opportunities where knowledge use in practice is 'observable'.

A formal letter of invitation from the principal investigator and the provincial site lead investigator will be sent to the administrator of the selected site to invite participation. Upon acceptance of the invitation, the provincial site lead investigator and respective research associate will arrange to meet with key people at the site. We will then negotiate the best means to successfully achieve access, implement adequate information sessions, disseminate printed study information, and schedule data collection.

We will collect data as follows:

1. Non-participant observation: Researchers will spend time in sites in a non-participant observer role.

2. Staff interviews: Formal and informal interviews with key informants including directors, care managers, allied health providers, registered nurses (RNs), and licensed practical nurses (LPNs) (approximately 10 to 12 per facility) plus 12 to 15 healthcare aides/facility.

3. Family interviews: Within each facility, we will assess the potential for up to three family caregivers, who regularly visit their loved one in the nursing home, to participate in an interview about their experiences of being a caregiver in the particular facility. They will be selected with the guidance and recommendation of the care manager and the method of approach will be tailored according to recommendations of the facility staff.

4. Staff focus groups: We will try to hold a staff focus group in each of the comprehensive and focused case study sites. In our experience, it is very difficult to arrange focus groups in nursing homes because of limited staff availability, therefore we will use naturally-occurring meeting groups to maximise the potential for participation.

#### Phase two

In phase two sites (up to two per province) will be purposively selected from the list of facilities not involved in the comprehensive case studies. Selection will take place following completion of data analysis for the comprehensive case studies and after emerging theory and/or set of themes has been inductively derived. At least one rural facility in Saskatchewan will be involved in phase two to ensure that any major differences between urban and rural facilities can be described.

The major driver for site selection in this second phase of the study will be the need to test emerging theories and explore similarities and differences about organizational context identified in the comprehensive case studies. Depending on the themes that emerge in the major case studies, key questions will be asked of the preliminary data from project one [2]. Answers to these questions, in combination with the emergent themes and theories, will be pivotal in making these site selections.

In contrast to the comprehensive case study sites, only one month will be spent collecting data in focused case study sites. This will enable a focused period of participant observation, interviews, and focus groups to be conducted, and the most evolved version of theory to be evaluated.

If the process of soliciting family's perspectives from the comprehensive case studies proves workable and fruitful, then one interview will be conducted in each of the focused case studies with three family members.

### Inclusion/exclusion criteria

The inclusion and exclusion criteria that will apply for the comprehensive and focused case studies as well as for the staff and family/caregivers are detailed in Table 1.

**Table 1:** Inclusion and exclusion criteria

	Inclusion	Exclusion
Comprehensive case studies	Facility:	Facility:
	1. One of the 30 urban facilities being sampled in project one	1. Undergoing (or expected to undergo) a degree of organizational flux within the proposed five-year lifespan of the TREC program
	2. While not prescriptive, we will consider the following factors in selecting this facility:	
	a. interest among the senior management to grant access for the study	
	b. willingness of care managers, healthcare aides, and other staff to be observed and interviewed for the study	
	c. practical issues, such as travel time to the facility	
	d. opportunities to maximize data collection by the existence of venues where knowledge use in practice is discussed and therefore 'observable'	
	e. availability of written documents that guide the use of knowledge in practice	
Focused case studies	Facility:	Facility:
	1. One of the 30 facilities being sampled in project one	1. Participation in the comprehensive case study
Family/caregivers	1. Regularly visit their loved ones	
Staff and physicians	1. Staff employed by facility for at least three months	1. Student
	2. Staff who have worked a minimum of 6 shifts per month	2. Physicians not currently seeing residents
	3. Staff can identify a unit where they work most of the time	3. Residents or Medical students
	4. Staff able to read and write English	4. Academic staff
	5. Physicians self-describe 30% of their practice as being seniors in long-term care	5. Clinical instructors whose primary role is supervising students
	6. Physicians have seen residents in the facility for at least three months	

### Data collection

#### Non-participant observation

A primary source of data will be non-participant observation in the long-term care facility, including observation in resident care areas and at a variety of meetings. General observations within units of the facility will provide a description of the daily work patterns that provide the organizational context for knowledge use. At the onset of phase one (the comprehensive case studies) the research associates will spend at least one month in each facility to observe natural activities, practices, and interactions among staff and residents. Relevant meetings will also be attended. In phase two (the more focused case studies) non-participant observation will take place over a shorter period.

These general observations will be broadly guided by Spradley's [36] nine dimensions of observation, which include: space, actors, activities, objects, acts, events, time, and goals and feelings. Particular attention will be paid to observing knowledge use with respect to indicator conditions: falls, pain, dementia behaviors, and quality of life. These indicators were identified by our decision-making partners as priority areas for long-term care facilities in Western Canada and will also be examined using numerical data in project one [2]. The team is also interested in focusing on skin care practice, which during preliminary data collection in sites also appears to be an important indicator. Observations will be recorded in researcher field notes that will subsequently be transcribed and imported into a qualitative data management/analysis software program.

#### Interviews

In addition, formal and informal interviews will be conducted with key informants to clarify factors in the setting that may be influencing knowledge use and characterizing context. Interviews will take place with key informants at various levels of the organization. These interviews will be conducted using interview guides that elicit views on key elements of organizational context (*e.g*., resources, organizational structure, leadership, team work). The actual format of the interviews will be tailored to the individual being interviewed. For example, the director of the facility will be an important informant on how various historical/critical events (*e.g*., implementation of new policies in the health region) have influenced knowledge use in practice. They will also be important sources of information about governance and resources, such as budgetary issues including pay and incentives, access to training opportunities for frontline staff, the status of information and knowledge management systems in the facility, and mechanisms for providing individuals' and groups' feedback on their performance. Frontline managers will be important sources of data with respect to teamwork issues on their unit, such as division of labor, pressure groups, and relationships between groups (*e.g*., day and night staff). Individual healthcare aides will also be interviewed to elicit their understanding of 'how things are done around here,' or the context of their unit and work shift for knowledge use. The questions to be posed to the managers will be driven by issues identified in the organizational literature as to the important influences on knowledge use in practice. On the other hand, the development of the interview guide for the healthcare aides will evolve as on-site observations are made to ensure that the questions asked will be framed in the language currently in use on their particular unit(s). During the first month of data collection, informal discussions will be undertaken simultaneously with the non-participant observation, which will enable any issues that arise in observation to be explored in more depth during formal interviews.

#### Documents

Documents will also be an important source of data. Documents that describe the organizational structure and staffing patterns will be summarized and described in field notes. Written policies and procedures, particularly with respect to the indicator conditions (falls, pain, dementia behavior, quality of life, skin care), will be gathered and analyzed to determine the knowledge products available for use in the facility. Informal materials, such as postings on bulletin boards, kardexes where information about care plans for individual residents often reside, and materials found in the staff resource room will be recorded in field notes. Where appropriate and with the consent of the facility, digital photographs of the physical layout of the unit or facility (not including residents, visitors, or staff) will be taken to provide information about the physical environment/context. The actual use of such documents on a daily basis as care is provided and as critical incidents occur, will also be recorded in the written field notes. Document data collection will take place on an ongoing basis during field visits.

#### Family perspectives

To better understand the effect of knowledge use within each of the facilities on the 'customer' of care, three family caregivers who regularly visit their loved one in the nursing home will be approached to participate in the study. These family caregivers will be selected with the guidance and recommendation of the unit manager, or if a caregiver expresses an interest in being involved during periods of observation. The family caregivers will be asked to take part in an interview in which we will explore their experiences and perspectives of being a caregiver in the particular facility. If this approach appears to be workable, then one interview will be conducted in each of the focused case studies with three family members. These interviews will provide another dimension for development of the theory.

#### Focus groups

Finally, after the major period of observation and interviewing is complete in phase one, and the data analysis has revealed major themes about elements of organizational context that influence knowledge use in practice, a focused discussion with staff members at naturally occurring meetings will be conducted. The intent is to present the themes along with their definitions and some descriptive examples so that the participants can comment on their perceptions of the accuracy of the interpretation made by the investigators. This is an important last step to provide for clarification, verification, and elaboration of the themes. Over a period of time, this process will evolve so that the participants in the final discussion groups during phase two will be providing feedback on the most evolved version of the theory, allowing for in-depth discussion of the circumstances under which certain factors are more or less influential in knowledge use. This process will also allow the investigators to truly embed the emerging theory in the language that is actually used within long-term care facilities to describe knowledge use in practice, thereby aiding in the dissemination and clarity of the findings for all levels of staff in these organizations.

#### Training for research associates

We are conscious of the need to provide relevant training and support for the qualitative fieldworkers. The leaders of the project will ensure that each person involved in the data collection process (primarily the research associates):

1. Is confident in designing interview and focus group schedules.

2. Can undertake content analysis of interview and focus group data.

3. Is aware of the ethics and sensitivities of conducting observation in practice settings.

4. Can take good field notes and analyze them.

5. Can interview and run focus groups effectively.

6. Is aware of the need for project management skills.

7. Can communicate the results of the analysis of qualitative data in written and verbal form.

8. Can work effectively as a team member with the other research associates and investigators.

The project leaders are experienced in the teaching and practice of these methods and have access to training materials to support the proposed approach. In addition, the research associates will receive technical training in the use of data management and communication systems (Nvivo, Elluminate, and the web-based learning management system) to facilitate the conduct of the project.

#### Analysis

Consistent with case study methods, each case is regarded as a 'whole study' in which convergent evidence is sought and then considered across multiple cases. As such, a pattern-matching logic, based on explanation-building will be used as a data analysis framework [32]. This strategy will allow for an iterative process of analysis across sites and will enable an explanation about organizational context and knowledge use to develop over time. It will be important to ensure that data analysis reflects the variety of data sources and the potential insight that each could offer in meeting the aims of this study. Therefore, data from each site will be analyzed within the data set to derive key themes, and then these themes will be considered in conjunction with the findings from the other data sources. Additionally, analysis will be conducted within sites and then, in order to enable conclusions to be drawn for the study as a whole, findings will be summarized across sites to assist in explanation and theory building. Data will be managed in NVivo [37].

The analysis process necessarily involves significant interaction between the investigators and the research associates who are actually making the observations and conducting the interviews. A plan has been developed to ensure that this interaction is timely, regular, and purposeful. To provide for multiple perspectives on the meaning of the data and how to label and define emerging themes, a key feature of this type of interpretive analysis is to provide for several individuals to read and interpret the field notes and interviews along with the individuals who have done the data collection. The investigators involved in this study all have significant previous experience in this analysis approach, and will ensure that data collection remains focused on defining the emerging explanation of how organizational context influences knowledge use. In this way, data collection continues only to the point of theoretical 'saturation' (that is, dense and rich explanation) so as to reduce burden to participants in the study.

#### Ethical issues

Ethical approval for this project has been obtained from the appropriate university ethics boards: Universities of Alberta, Calgary, Manitoba, Saskatchewan, and Regina. Relevant operational approvals will be obtained accordingly.

#### Access to sites

Access to sites was described above. Sites are free to withdraw at any stage of the research.

#### Consent

Participation is voluntary. Consent will be negotiated at several levels. First, the facility itself will be asked to consider the request for their inclusion in the study, and written consent to make general observations in designated areas and to analyze written documents will be requested from the official authorities of the facility. Second, a principle of consent by exclusion will be followed when observing in general resident care areas. That is, the consent of staff on each shift where observations will take place will be verbally negotiated; anyone who is not comfortable with being included will not be observed. No families, residents, or staff will be observed without their consent. Third, staff will be told that at any time they can ask to see the notes that the observer is recording if they wish to, so that they can clarify any inaccuracies, or they may ask that the observer stop recording. All such requests will be respected. No observations will occur in residents' rooms, but observations of residents will occur in common areas, such as hallways and lunch rooms. In any case where observations may occur in common areas, we will negotiate this with staff and residents (if able) or family if not. Printed material in the form of brochures and posters will be available for residents, visitors, and staff to explain the study's purposes and data collection approaches.

Written consent will be obtained for individual interviews. These consents will be negotiated in advance, and a convenient time will be arranged for the interview to occur in a quiet, non-threatening setting. Our experience from feasibility testing in project one [2] has been that the healthcare aides find the process of informed consent to be positive.

#### Anonymity

Participants in this project will not be anonymous to the frontline data collectors. In addition, audio-recordings may contain information that enables identification of the participants. All audio-recordings will be stored centrally on a secure server and will remain identifiable by codes assigned by the researchers. Back-up copies of audio-recordings will be stored on computer disks, which will be kept in a locked cabinet. Code legends will be stored securely and separately from the audio-recorded data. De-identification will occur at the point of transcription and the process by which this occurs will be decided upon by the TREC data committee and project investigators, in consultation with the research associates. All transcriptionists will sign a confidentiality oath and all data in this study will be held confidentially.

#### Harm

This study and the observations are being conducted in settings with vulnerable populations, *i.e*., long-term care residents. During the course of the observational work investigators, research associates and/or staff may observe instances of unsafe or unethical practice. If they arise, we will deal with these situations on a case-by-case basis in accordance with professional guidelines and facility procedures.

#### Burden

We will negotiate, and be flexible with, the times of data collection so as to avoid any untoward impact on the operations of the facility. As a token of our appreciation, we will provide the facility with refreshments and participating staff with a token gift (*e.g*., a coffee certificate).

## Discussion

This study has been designed to provide a rich picture and robust explanation of the way that organizational context mediates the use of knowledge in practice over time in long-term care facilities. As TREC as a whole has been designed to explore the unknown as well as build on what is already known; the robustness of that explanation will be partly dependant on how well project one [2] is integrated with this project, both practically and theoretically. During the conduct of this project, the intention is to continually consider the emerging findings from survey data collected in project one [2] and reflect on their implications for data collection and analysis in this study, and vice versa. Practically, this will require effective communication and the development of an integrated project timetable identifying critical junctures when these assessments and reflections can be made. Additionally, integration will require that TREC investigators maintain a clear theoretical drive within individual projects and theoretical thrust across the entire program of research, ultimately enhancing validity [38].

There will be a number of additional challenges associated with the running of this study. We have a geographically dispersed team and study sample that comes from different Canadian provinces and countries. To ensure that optimal communication occurs between the investigators and researchers a number of mechanisms have been implemented. These include a communication strategy comprising regular updates to describe the progress and achievements of each of the projects, regular teleconferences, the creation of secure virtual spaces for sharing data and information and for contributing to data analysis, and face-to-face meetings at critical points of the project. In addition, a project manual detailing clear procedures for sharing and handling data has been developed to ensure data collection and handling is conducted in a manner in which the security, integrity, and quality of the data is maximized.

Retention of the sites that consent to participate in this study poses a serious challenge. The overall TREC program of research is being conducted over five years. During this time it is likely that the facilities will experience a number of changes. We are acutely aware that some facilities may choose to withdraw from the study at some stage. Furthermore, we are mindful of the potential to overburden the staff and facility management during data collection. We will therefore strive to avoid this by maintaining open communication with facilities and encouraging them to articulate any concerns regarding the conduct of the study. We will also be developing feedback mechanisms with facilities so that they can benefit from the information being gathered by the researchers. In addition, we will be seeking to capture if, and how, the feedback has influenced practice. Detecting change in practice in response to the feedback, as opposed to practice change resulting from other activities or interventions, will require meticulous attention to detail during qualitative data collection and analysis.

The complex nature of this study and the process of qualitative data collection and analysis necessitate recruitment of highly skilled and experienced personnel. Because of these requirements, attracting appropriately qualified personnel has presented a significant challenge. Research associates have been appointed into the role, and the retention of these individuals for the duration of the study is a high priority. We will actively work to provide these individuals with opportunities to develop new skills and to engage and stimulate them intellectually in the generation of new knowledge throughout the project.

Conducting research in the real-world setting requires patience and understanding, as well as the capacity to be flexible during data collection phases. We recognize that the day-to-day work of the healthcare providers we will be encountering can be stressful and unpredictable. Hence, data collection may not always go according to plan. Furthermore, changes within facilities may have implications for the study as a whole and will need to be carefully documented and their implications analyzed in terms of the conclusions that are drawn from the study.

### Limitations

This study will be limited to three provinces in Canada; hence generalizing findings beyond this context should be exercised with caution, and so readers will have to consider the finding's theoretical transferability. We will purposively select facilities in order to provide a representative sample of long-term care facilities across the three Prairie Provinces; the willingness of invited facilities to participate will determine the extent to which we are successful in this endeavor.

The trustworthiness of data collected in the real world, which demands flexibility and adaptability, may be questioned [39]. To enhance the trustworthiness of the data, we will provide regular, ongoing training for, and work closely with, the research associates throughout the data collection and analysis phases. In doing so, we will encourage constant reflection upon, and comparison between the themes as they emerge from the case studies. In addition, we will actively employ reflexivity and use research team debriefing opportunities to identify and help overcome potential sources of researcher bias [39]. Data triangulation is being used to capture a comprehensive picture of organizational context and its mediating effect on knowledge translation. This approach will help to illuminate our understanding of organizational context and knowledge use, as well as maximize the strength of the conclusions that are generated [40]. A detailed audit trail will be maintained throughout the study to enable retracing of analysis and ongoing reflection upon theory development and testing.

## Competing interests

The authors declare that they have no competing interests.

## Authors' contributions

CAE is the principal investigator for the TREC research program. She conceived the program and its design, secured its funding, and is providing the leadership and coordination for the program. LD is the lead investigator for project two. LD, SD and JR-M designed project two and participated in securing grant funding. JR-M and AH made the major contribution to writing the final manuscript; LD, SD, DM, NS provided commentary to drafts. All authors read and approved the final submitted manuscript.
